# Social support, positive caregiving experience, and caregiver burden in informal caregivers of older adults with dementia

**DOI:** 10.3389/fpubh.2023.1104250

**Published:** 2023-01-25

**Authors:** Marta Nemcikova, Zuzana Katreniakova, Iveta Nagyova

**Affiliations:** ^1^Department of Social and Behavioural Medicine, Faculty of Medicine, PJ Safarik University in Kosice, Kosice, Slovakia; ^2^Slovak Public Health Association - SAVEZ, Kosice, Slovakia

**Keywords:** dementia, informal caregivers, caregiver burden, social support, caregiving experience

## Abstract

**Introduction:**

Dementia is currently one of the major causes of disability and dependency among older adults worldwide. Cognitive dysfunction, neuropsychiatric symptoms, somatic complaints, and functional impairment fundamentally affect not only a person living with dementia (PLwD), but also his/her informal caregiver(s), often resulting in a high caregiver burden. A number of variables, including the caregiver's sociodemographic characteristics, the clinical characteristics of PLwD, social support, and the caregiver's personal resources determine the caregiver's burden.

**Objectives:**

The aim of this study was to investigate the associations of caregiver burden in informal caregivers of PLwD with perceived social support, positive caregiving experience, and applying therapeutic communication methods.

**Methods:**

The data were collected from September 2021 to February 2022 among 115 “PLwD—informal caregiver” dyads in the community settings in Slovakia. Measures included the Zarit Burden Interview (ZBI-12), the Oslo Social Support Scale (OSSS-3), the Positive Aspects of Caregiving Scale (PACS), and two questions on applying therapeutic communication methods—reminiscence and validation according to Naomi Feil. The Short IQCODE was used for assessing cognitive decline in PLwD. Pearson's and Spearman's correlations, *t*-tests, Chi-square, ANOVA, and linear multiple regression analyses were used to analyze the data (IBM SPSS 27).

**Results:**

The mean age of informal caregivers was 54 ± 12.4 years (81.7% of women) and the mean caregiving duration was 4.8 ± 4.8 years. The mean age of PLwD was 80.5 ± 8.3 years (73.0% of women) and their Short IQCODE mean score was 4.1 ± 1.0. Lower caregiving burden was significantly associated with higher perceived social support (β = 0.33, *p* < 0.01), with higher positive caregiving experience (β = 0.33, *p* < 0.01), and higher caregiving intensity (β = 0.24, *p* < 0.05) among informal caregivers of PLwD. The associations between caregiver burden and applying two therapeutic communication methods were not significant.

**Conclusions:**

Implementing psycho-social and educational public health interventions focused on strengthening social support and maintaining positive perceptions of caregiving can help reduce the increased risk of caregiver burden in informal caregivers of older adults with dementia.

## Introduction

Dementia is one of the major public health challenges in older adults and is most commonly caused by Alzheimer's disease (1). In 2015, almost 50 million people worldwide were living with dementia (2). According to Alzheimer Europe (3), the incidence of dementia syndrome is alarming and is estimated to double by 2050 mainly among people over the age of 65. This also applies to Slovakia, where the estimated number of dementia patients is 62,495 representing 1.15% of the population. In 2018, the estimated prevalence of dementia was highest among men from 80 to 84 years (3,370 persons) and women from 85 to 89 years of age (9,740 persons) in Slovakia (3).

A person living with dementia (PLwD) is fundamentally affected by a progressive and irreversible cognitive deficit (4), with a wide range of neuropsychiatric symptoms including delusions, hallucinations, insomnia, somatic complaints, and functional impairments. However, dementia syndrome affects not only the PLwD but also relationships with informal caregiver(s), i.e., their relatives, partners, or close friends (5). During the disease progression, changes in thinking and behavior are not only the result of pathology in the brain but are sometimes more complex expressions of feelings, experiences, and personality, which in many cases results in communication misunderstandings (6). Informal care presents a huge challenge for families and friends of PLwD (1).

“A multidimensional response to physical, psychological, emotional, social, and financial stressors associated with the caregiving experience” was defined as a caregiver burden [(7); p. 119]. While the objective caregiver burden refers to the amount of time spent on caregiving and the tasks that are performed, the subjective burden indicates how the informal caregivers experience performing their caregiving tasks and may refer to the physical, psychological, emotional, social, and/or financial consequences of caregiving (8, 9). Studies on caregiver burden related to caring for PLwD highlight its negative effect on physical (10) as well as on mental health (11), (12). Moreover, low levels of knowledge, health beliefs, and attitudes toward health (risk reduction) play also an important role in participating caregivers in health-promoting and disease-preventive actions which can decrease the negative impact of caregiving burden on their health (13–15). Due to its multidimensional construction caregiver burden is seen as a complicated concept that is not always static (16).

According to previous studies, social support is recognized as one of the mediators of caregiver burden (17). Two dimensions of social support are distinguished in the literature, perceived and received social support. Perceived social support is the perception of the extent and quality of support an individual receives from his or her social network. It is fulfilled through social relationships, when, thanks to affective support, it helps to reduce the psychological burden of the caregiver of a PLwD (18). It is therefore essential that the individual family members of the PLwD support each other. Especially when reorganizing their lives in crises are very emotionally stressful. Clear and open communication during this process strengthens resilience, which in turn contributes to family adaptation (19). Received social support is defined as an objective quantification of the help and support people receive from their social network (18, 19). Connors et al. (20) reported that those who use a home care professional service have a lower caregiver burden than to those who do not. Contrary to this, Wang et al. (21) found that those clients who used outreach services were more dependent in daily living activities and therefore their informal caregivers had a higher burden. The results of a meta-analysis by Del-Pino-Casado et al. (22) comparing 46 studies on perceived social support and 16 studies on received social support confirmed the existence of a negative relationship between perceived social support and subjective caregiver burden and a very small negative relationship between received social support and subjective caregiver burden. Research has also found that perceived social support is a stronger predictor of individual wellbeing than received social support because it is closely related to personality traits such as optimism and self-confidence (23, 24).

The nature of the caregiving experience can be positive or negative. If the caregiving experience is perceived positively, it can lead, for example, to increase self-esteem or improve relationships. One of the positive aspects of caregiving is that the informal caregiver feels useful and satisfied (25). Caregiving is also perceived as less stressful when the relationship between caregiver and care recipient is filled with love (26) and when the caregiver's resources and needs are balanced (27). A positive perception of caregiving is related to life satisfaction (28).

It is already known that for the development of social support and strengthening of a positive caregiving experience psychosocial interventions oriented toward building appropriate coping strategies can be effective (29). In addition, these interventions can focus also on psychoeducation in the area of communication with PLwD, for example by applying the method of reminiscence (30) or validation (31). Both techniques are aimed at neuropsychiatric symptoms of dementia and belong to emotion-oriented care in dementia (32, 33). Validation can also have a stress-reducing potential (34). According to available literature, the relationship between the caregiving burden and applying validation has not yet been investigated in informal caregivers of PLwD.

Despite the fact that the caregiver burden is related to the negative or positive feelings and perceptions of the caregiver associated with providing care, there is more literature on the negative impact of caregiving (16). Therefore, by using a theoretical model-based approach (e.g., stress-appraisal models of caregiver wellbeing) more complex mediating and moderating relationships between caregiver wellbeing and burden can be uncovered (35). The aim of this study was to investigate the associations of caregiver burden in informal caregivers of PLwD with perceived social support, positive caregiving experience, and applying therapeutic communication methods.

## Materials and methods

### Sample and procedure

Data were collected from September 2021 to February 2022. Implementation of the cross-sectional study was approved by the Ethics Committee of the Faculty of Medicine, Pavol Jozef Safarik University in Kosice (No. 1N/2021). The inclusion criteria were age over 18 years and the status of an informal caregiver of a PLwD. Participation was voluntary, anonymized, and based on expressing informed consent for inclusion in the study. An opportunity to participate in the research was advertised through online media–websites and social networks. All 12 Slovak specialized outpatient social care facilities for individuals with dementia were also invited *via* e-mail. Another 100 questionnaires were distributed in printed form by regular mail through one healthcare provider and seven providers of home social services in the Kosice self-governing region. Filling out the self-administered questionnaire took about 15–20 min. The questionnaires for informal caregivers included also data about their PLwD. Given that 20 respondents did not meet the condition of being an informal caregiver of a PLwD, 115 of the 135 study participants were included in the data analyses.

### Measures

The selection of self-assessment instruments was based on an adaptive version of the stress-appraisal model of Chappell and Reid (36) expanded with psychosocial interventions (reminiscence, validation) and controlled variables (sociodemographic and clinical variables of the informal caregiver).

*The Zarit Burden Interview* (ZBI-12) (37) was designed for the assessment of subjective caregiver burden (38) and consists of 12 items. Each item is assigned a 5-point Likert scale ranging from 0 to 4, where 0 means never and 4 is almost always. The total score is the sum of all points for each item. A higher score indicates a higher level of burden. Scores range from 0 (low burden) to 48 (high burden), with a cut-off score ≥17. The Cronbach's alpha value of the ZBI-12 in our study was 0.90.

For measuring *the objective caregiver burden*, data on the caregiving duration were collected as the total years of care provided. The caregiving intensity was also measured as the extent of care provided in hours per week at three intervals: ≤ 8, 9–39, and >40.

*The Oslo Social Support Scale* (OSSS-3) (39) is a three-item questionnaire that includes questions about the number of close people (confidants), the feeling of concern or interest in other people, and the relationship with neighbors. The total score ranges from 3 to 14, with higher values representing a higher level of perceived social support. The social support score can be divided into three categories: weak (OSSS-3 = 3–8), medium (OSSS-3 = 9–11), and strong (OSSS-3 = 12–14) social support (40). The Cronbach's alpha value of OSSS-3 in our study was 0.63.

For measuring *the received social support*, information about the type of care and the degree of cooperation provided by formal social and health care service providers based on the current national legislation were collected.

*The Positive Aspects of Caring Scale* (PACS) (41) was used for assessing caregivers' positive beliefs and attitudes regarding the value of the caring role. The PACS consists of 6 items, and the answers use a 4-point scale (from 1 = strongly disagree to 4 = strongly agree) with a total score ranging from 6 to 24. A higher score means that the caregiver has a higher positive attitude toward their own role as a caregiver. The PACS has a good internal consistency with a Cronbach alpha coefficient of 0.89 (41). The Cronbach's alpha value of PACS in our study was satisfactory at 0.88.

Applying therapeutic communication methods was measured by the two close-ended questions “*Do you yourself apply the concept of reminiscence? Do you yourself apply the concept of validation according to Naomi Feil?*” with 1 = yes and 2 = no answers.

*The Informant Questionnaire on Cognitive Decline in the Elderly* (IQCODE) (42) is a widely used measurement tool designed for dementia screening, especially in cases where direct cognitive testing is not possible due to low levels of education and literacy, acute illness, lack of interest, or death of individuals. It is used for measuring cognitive decline from the premorbid level using the reports of an informant (43), who in our study was an informal caregiver. We used the short version of the IQCODE, which contains 16 items that assess changes over the past 10 years. Each response is scored from 1 (much better) to 5 (much worse). When calculating the scores, the answers to all questions are added up and the sum is divided by the number of items. The Cronbach's alpha value of the Short IQCODE in our study was 0.98.

### Statistical analysis

Frequencies, means, and standard deviations were calculated to describe the study sample and the assessment measures. Pearson's and Spearman's correlations, *t*-tests, Chi-square, and analysis of variance (one-way ANOVA) were used to analyze the associations of subjective caregiver burden (ZBI-12) with age, gender, education, social support, caregiving experience, communication methods, and caregiving intensity. Finally, after checks for multicollinearity, linear multiple regression analysis (step method) was conducted to analyse the associations between caregiver burden (ZBI-12), controlled for age, gender, education (Model 1), reminiscence, validation (Model 2), positive caregiving experience (Model 3), social support (Model 4), and caregiving intensity (Model 5). Statistical analyses were performed using the IBM SPSS version 27.0 software. We used Power analysis to determine the sample size and statistical power of the results (44). *Post hoc* power analysis for linear multiple regression in a sample of *N* = 115 respondents reached the level of 0.83 with a medium effect size at the level of statistical probability alpha = 0.05. The results of statistical tests were considered statistically significant at *p* < 0.05.

## Results

### Characteristics of informal caregivers and PLwD

The research sample consisted of 230 respondents, of which 115 were informal caregivers and 115 were PLwD as receivers of informal care. Their socio-demographic characteristics are shown in Table 1.

**Table 1:** Characteristics of the study sample—informal caregivers (*N* = 115), persons living with dementia (*N* = 115).

**Characteristics**	***n***	**%**	**Mean**	**SD**	**Range**
**Informal caregivers**					
**Gender**					
Male	21	18.3			
Female	94	81.7			
Age			54	12.4	26–84
**Relationship to care receiver**					
Spouse	18	15.7			
Daughter/son	60	52.2			
Granddaughter/grandson	6	5.2			
Other	31	27.0			
**Marital status**					
Single	21	18.3			
Married	69	60.0			
Divorced	13	11.3			
Widowed	4	3.5			
Other	5	4.4			
Caregiving duration (years)			4.8	4.8	0.2–21
**Caregiving intensity (hours per week)**					
≤ 8	19	16.5			
9–39	32	27.8			
>40	64	55.7			
**Education**					
Elementary	22	19.1			
Secondary	28	24.3			
University	65	56.5			
**Occupation**					
Employed	80	69.6			
Retired	25	21.7			
Unemployed	1	0.9			
Other	9	7.8			
**Living area**					
Urban	81	70.4			
Rural	34	29.6			
**PLwD**					
**Gender**					
Male	31	27.0			
Female	84	73.0			
Age			80.5	8.3	51–98
**Dementia type**					
Alzheimer's disease	55	49.1			
Other dementia	57	50.9			
**Form of received social care**					
Home care	24	20.9			
Daycare	15	13.0			
Resident	4	3.5			

The percentage of missing values for each variable were: Informal caregivers—gender 0%, age 0%, relationship to care receiver 0%, marital status 0%, caregiving duration 3.5%, caregiving intensity 0%, education 0%, occupation 0%, living area 0%; PLwD—gender 0%, age 0%, dementia type 2.6%, form of received care 0%.

In our research sample, informal care was mainly provided by women (81.7%). The mean age of informal caregivers was 54 ± 12.4 years ranging from 26 to 84. Almost three-quarters of respondents lived in cities (70.4%) and 60.0% were currently married. More than half had a university education (56.5%) and were employed (69.6%). The most prevalent type of relation of informal caregivers to PLwD was being a son or daughter (52.2%), and the second most relevant was a spouse (15.7%). The remaining were persons in a different relationship with the care receiver, i.e., daughter-in-law, sister, nephew, cousin, or acquaintance. The mean caregiving duration was 4.8 ± 4.8 years with a time variance from 2 months to 21 years. In terms of caregiving intensity, more than half of the respondents (55.7%) provided care for more than 40 h per week. Out of caring activities, the respondents most often declared guiding for medical examinations, helping with official and administrative issues, and supporting social activities (85.2%). More than three-quarters (79.1%) were involved in household care, food shopping, food preparation or delivery, and cleaning. More than half helped with self-care tasks (62.6%) and supervised home care and self-care tasks (60.0%).

The majority of PLwD were women (73.0%). Their mean age was 80.5 ± 8.3 years, ranging from 51 to 98. Almost half of the patients were diagnosed with Alzheimer's disease (49.1%), followed by vascular dementia (8.9%), Parkinson's disease (6.3%), and 3.6% of other dementias (mixed or alcoholic dementia). The remaining respondents did not know the exact type of dementia of PLwD. The cognitive level of PLwD was measured by the Short IQCODE and reached an averaged value of 4.1 ± 1.0.

Table 2 displays descriptive characteristics of the major variables assessed in the informal caregivers of PLwD. The total ZBI-12 mean sum score achieved was 17.9 ± 9.9. Low caregiver burden (ZBI-12 < 17) was found in less than half of the respondents (47.5%). The mean score of perceived social support was 8.9 ± 2.4. Strong social support was reported only by 17.4%. Received social support measured by the type of care and the degree of cooperation provided by formal care providers was low. Informal caregivers from our study provided care mostly by themselves without any professional help, but with the support of other family members. The PACS mean score was 17.9 ± 4.1. The concept of reminiscence was applied by 37.2% and the concept of validation according to Naomi Feil was used by only 22.4% of informal caregivers.

**Table 2:** Mean scores for the assessment measures.

**Variable**	***n***	**%**	**Mean**	**SD**	**Range**
**Caregiver burden**					
Low (ZBI-12 < 16)	48	47.5	17.9	9.9	0–47
High (ZBI-12 ≥ 17)	53	52.5			
**Social support**					
Poor (OSSS-3 = 3–8)	48	44.0	8.9	2.4	4–14
Moderate (OSSS-3 = 9–11)	42	38.5			
Strong (OSSS-3 = 12–14)	19	17.4			
**Caregiving experience**					
PACS	100		17.9	4.1	7–24

ZBI-12, the Zarit Burden Interview; OSSS-3, the Oslo Social Support Scale; PACS, the Positive Aspects of Caregiving Scale.

### Associations of caregiver burden with social support, positive caregiving experience, and therapeutic communication methods

Pearson's and Spearman's correlations of subjective caregiver burden with age, gender, education, applying communication methods, social support, caregiving experience, and caregiving intensity were calculated and are presented in Table 3. Lower caregiver burden was significantly associated with a higher perceived social support (*r* = −0.52, *p* < 0.01), with a higher positive caregiving experience (*r* = −0.51, *p* < 0.01), with a higher caregiving intensity (*r* = −0.26, *p* < 0.05) and with a lower education (*R* = 0.26, *p* < 0.05). The Chi-square test was used to estimate the association between subjective caregiver burden and applying validation according to Naomi Feil as a therapeutic communication method. The result was not significant (Table 4). Additionally, an ANOVA test was used to examine the objective caregiver burden by means of caregiving intensity in relation to the subjective caregiver burden. The results were also not significant (*F* 1.591, *p* = 0.209).

**Table 3:** Correlations of subjective caregiver burden with caregivers' age, gender, education, applying communication methods, perceived social support, positive caregiving experience, and caregiving intensity in informal caregivers of PLwD.

	**Caregiver burden**
**Independent variables**	**ZBI-12**
Age	−0.02
Gender	0.15
Education	0.26^*^
Reminiscence	0.05
Validation	0.14
PACS	−0.51^**^
OSSS-3	−0.52^**^
Caregiving intensity	0.26^*^

ZBI-12, the Zarit Burden Interview; OSSS-3, the Oslo Social Support Scale; PACS, the Positive Aspects of Caregiving Scale.

^*^p < 0.05.

^**^p < 0.01.

**Table 4:** Associations between subjective caregiver burden with applying therapeutic communication methods (*t*-tests, Chi-square), and with objective caregiver burden (One-way ANOVA).

**Caregiver burden**			**Differences between groups**	**Caregiver burden**		**Differences between groups**
	**ZBI-12**			**ZBI-12**<**16**	**ZBI-12** ≥**17**	
	***n*** **(%)**	**Mean** ±**SD**		***n*** **(%)**	***n*** **(%)**	
Reminiscence	78		ns			
Yes	29 (37.2%)	18.8 **±** 9.9		-	-	-
No	49 (62.8%)	19.4 **±** 9.7				
Validation	76		ns			ns
Yes	17 (22.4%)	17.2 **±** 8.8		8 (47.1%)	9 (52.9%)	
No	59 (77.6%)	20.6 **±** 9.6		20 (33.9%)	39 (66.1%)	
Caregiving intensity	115		ns			
≤ 8 h per week	19 (16.5%)	16.0 **±** 8.8				
9–39 h per week	32 (27.8%)	15.7 **±** 8.4		-	-	-
>40 h per week	64 (55.7%)	19.4 **±** 10.7				

ZBI-12, the Zarit Burden Interview.

In Model 1, age (β = 0.33, *p* < 0.05) and education (β = 0.36, *p* < 0.01) were significantly associated with ZBI-12 and explained 12% of the total variance in ZBI-12 (*p* < 0.05). When reminiscence and validation were added (Model 2), age and education remained significant (*p* < 0.01). Reminiscence and validation were not significantly associated with the ZBI-12. After adding PACS (β = −0.47, *p* < 0.001), the explained variance in ZBI-12 increased from 15 to 33% (Model 3). After adding OSSS-3 (β = 0.34, *p* < 0.01), the explained variance in ZBI-12 increased to 42% (Model 4). The association between age and ZBI-12 (Model 1–Model 4) was no longer significant when caregiving intensity (β = 0.24, *p* < 0.05) was added to Model 5. The associations between education (β = 0.23, *p* < 0.05), PACS, OSSS-3 (β = 0.33, *p* < 0.01), and ZBI-12 were significant and the final model (Model 5) explained 47% of the ZBI-12 total variance (Table 5).

**Table 5:** Linear multiple regression analyses between subjective caregiver burden and socio-demographic characteristics (Model 1), applying therapeutic communication methods (Model 2), caregiving experience (Model 3), social support (Model 4), and caregiving intensity (Model 5).

		**Caregiver burden**	
		**ZBI-12**	
	β	**adj** *R*^2^	**smc**
**Model 1**			
Age	0.33^*^	0.12	^*^
Gender	0.13		
Education	0.36^**^		
**Model 2**			
Age	0.34^**^	0.15	ns
Gender	0.20		
Education	0.36^**^		
Reminiscence	0.15		
Validation	0.14		
**Model 3**			
Age	0.32^**^	0.33	^***^
Gender	0.17		
Education	0.27^*^		
Reminiscence	0.04		
Validation	0.03		
PACS	−0.47^***^		
**Model 4**			
Age	0.22^*^	0.42	^**^
Gender	0.09		
Education	0.20		
Reminiscence	0.05		
Validation	−0.02		
PACS	−0.35^**^		
OSSS-3	−0.34^**^		
**Model 5**			
Age	0.17	0.47	^*^
Gender	0.11		
Education	0.23^*^		
Reminiscence	0.06		
Validation	0.04		
PACS	−0.33^**^		
OSSS-3	−0.33^**^		
Caregiving intensity	0.24^*^		

ZBI-12, the Zarit Burden Interview; PACS, the Positive Aspects of Caregiving; OSSS-3, the Oslo Social Support Scale; β, beta; smc, significance of model change for the added variable(s).

^*^p < 0.05.

^**^p < 0.01.

^***^p < 0.001.

## Discussion

This study aimed to investigate the associations of caregiver burden in informal caregivers of PLwD with perceived social support, positive caregiving experience, and applying therapeutic communication methods.

A high caregiver burden was found in more than half of the informal caregivers of PLwD in our study sample. Previous studies have revealed that the prevalence of high levels of burden in informal caregivers of PLwD is about 20%, with a further 58% of caregivers at increased risk of distress (45). In our sample, more than half of the respondents had a university degree and were employed. Current trends predict a future increase in employment among informal caregivers, especially wives and daughters of older persons (46). The majority of informal caregivers in our sample were women (81.7%), similar to many other countries (26, 47). Compared to men, a higher burden was identified in women in the previous studies, while our study did not show gender differences in perceived caregiver burden. This result may also be caused by the low number of male participants included in our study.

We found a strong association between higher positive caregiving experience and lower subjective caregiver burden, even after controlling for age, gender, and education. Our findings and the findings of other authors (48) on significant associations of a lower subjective caregiver burden in informal caregivers of PLwD and with a higher positive caregiving experience are supported also by the study of (Brand et al. (23). They demonstrated an association between higher social support and higher levels of optimism, which had associations also with a sense of value, ability to care, and caregiver satisfaction. Similar associations of positive caregiving experience in informal caregivers of PLwD with both objective and subjective burden, avoidance coping, and perceived social support were found in Grover et al. study (49), in which other measurement tools were applied including the Burden Interview Schedule, the Social Support Questionnaire, and the Scale for Positive Aspects of Caregiving Experience.

Multiple linear regression analyses also identified a strong association between higher percieved social social support and lower subjective caregiver burden. Our findings regarding associations of a lower subjective caregiver burden in informal caregivers of PLwD with a higher rate of perceived social support are in line with the study of Yu et al. (48). The negative association between subjective caregiver burden (as measured by the ZBI) and perceived social support (as assessed by the Multidimensional Scale of Perceived Social Support) was demonstrated also by Putir et al. (50) on a larger sample consisting of 207 informal caregivers of PLwD aged 60 years and over and by Abdollahpour et al. (51).

Multiple linear regression analyses showed a weak but significant association between higher caregiving intensity and subjective caregiver burden. The study of Yu et al. (48) confirmed that a higher level of caregiving burden was significantly associated with a higher caregiving intensity. Controlling for the demographic variables of the caregivers, [Cao and Yang (9)] also found that subjective and objective dementia caregiving burdens were significantly associated. Furthermore, other authors (29) demonstrated that caregiving intensity has a non-linear relationship with caregiving burden. However, they investigated the response to the caregiving intensity with an open-ended question: “How many hours a day do you spend caring for a family member with dementia?” Based on responses from 637 respondents they found that if the hours of care reach a certain range, i.e., 14 h per day, the level of caregiver burden is high (measured by the ZBI-12), but for the range of care over 14 h per day, more hours of care meant a lower burden level. In our sample, where the caregiving intensity was measured at three intervals (≤ 8, 9–39, >40 h per week), more than half of the respondents provided care for more than 40 h per week, which corresponds to at least 5.7 h a day. The time spent in informal caregiving can be also considered and may limit informal caregivers in recreational or social activities (52), especially in the absence of opportunities for respite care. It is already known, that up to 41% of informal caregivers experienced an increase in caregiving burden due to the COVID-19 pandemic. Regression analysis results confirmed that factors such as the duration and intensity of caregiving were related to increasing caregiving burden during the COVID-19 pandemic in the Japanese study of Otobe et al. (53).

Our analyses did not show a statistically significant difference in the level of subjective caregiver burden between those who applied vs. did not apply the communication methods (reminiscence and validation according to Naomi Feil). As the validation has not yet been investigated in relation to the caregiver burden of PLwD as an independent intervention, the findings from multi-component psychosocial interventions (54) are available only, who investigated the caregiver burden and stress among 104 informal caregivers. While, the assumption about significantly decreased caregiver burden 1, 3, and 6 months post-intervention was not confirmed, the perceived stress was significantly reduced only after 6 months. Almost all respondents found validation particularly helpful in managing the behavioral challenges associated with dementia (54). These findings support the original hypothesis by Feil (1992 in Canon (55), p. 12) who stated that the validation “reduces stress and frustration in caregivers in social care facilities by improving interpersonal communication and developing a more meaningful relationship with the person with dementia.” Other three studies investigated the effect of multi-component and emotion-oriented interventions, including validation, and were devoted to increasing satisfaction and reducing stress symptoms in professional workers in health and social services (33, 56, 57). Another three studies (58–60) investigated the effects of validation, but none of them measured caregiving burden as a dependent variable. In general, many multicomponent studies reported a positive impact on caregivers especially in self-efficacy, burden, and depressive symptoms. However, the single-component psychoeducational interventions solely focusing on one strategy were rather not effective. This does not mean that these strategies should not be undertaken, but rather that they need to be embedded into more extensive interventions (61).

### Strengths and limitations, practice implications

Taking into account that caregiver burden may vary across caregivers caring for patients with different diseases, this is a novel and critical study that brings a more accurate understanding of the caregiver burden in relation to informal care for PLwD in the community setting. In some countries, there are relatively few studies focused on the investigation of caregiver burden related to the provision of dementia care. Moreover, these studies examine the caregiver burden from nursing (16) rather than the public health perspectives. The caregiving process is in general an inherently complex one and is in need of more research. Therefore, it would be helpful to conduct a longitudinal study to assess whether there are changes in caregivers' burden of PLwD over time and to identify variables and interventions that may positively influence those changes.

Several limitations of this study are important to consider. First, the study was cross-sectional in design; therefore, we cannot make any statements about causation and the findings might be susceptible to reverse causality. As our study consisted mostly of female participants more studies with a larger proportion of male caregivers are needed to shed more light on gender differences in caregiver burden. Another limitation of our study may be the time of data collection, which took place during the COVID-19 pandemic. This period was associated with several strict public health measures at the whole society level, such as the limitation of social contacts. Consequently, many caregivers experienced anxiety and fear of meeting other people regarding the person being cared for in order to protect them from a life-threatening infection (62). At the same time, we assume that some caregivers in older age could not participate in online data collection because they did not have available technical equipment or their digital literacy was low. All the data reported in this study relied on self-report, which is prone to bias. Also, the data collected about PLwD relied on information provided by the informal caregiver without objectification by clinical data. Despite these limitations, we see the strengths of our study in applying validated standardized instruments used in other countries as well as that both caregivers and PLwD data were collected.

## Conclusions

The significant associations of lower caregiver burden with a higher perceived social support and a higher positive caregiving experience reinforce the need for interventional public health programs aiming to decrease the overall imposed burden. It can be achieved through implementing psycho-social and educational interventions focused on emotions, especially in those caregivers with higher caregiving intensity. Such programs may strengthen social support, maintain positive perceptions of caregiving, and help to reduce the risk of caregiver burden in informal caregivers of PLwD.

## Data availability statement

The raw data supporting the conclusions of this article will be made available by the authors, without undue reservation.

## Ethics statement

The studies involving human participants were reviewed and approved by Ethics Committee of the Faculty of Medicine, Pavol Jozef Safarik University in Kosice (No. 1N/2021). The participants provided their written informed consent to participate in this study.

## Author contributions

MN: writing—original draft, conceptualization, and methodology. ZK: supervision, conceptualization, methodology, data curation, writing—review and editing, and funding acquisition. IN: supervision, methodology, data curation, formal analysis, visualization, writing—review and editing, and funding acquisition. All authors refined the manuscript for submission, read it, and approved the final version of the manuscript.
